# Development of prediction model for alanine transaminase elevations during the first 6 months of conventional synthetic DMARD treatment

**DOI:** 10.1038/s41598-023-39694-2

**Published:** 2023-08-09

**Authors:** Laura Kuusalo, Mikko S. Venäläinen, Heidi Kirjala, Sofia Saranpää, Laura L. Elo, Laura Pirilä

**Affiliations:** 1 Division of Medicine, Centre for Rheumatology and Clinical Immunology, University of Turku and Turku University Hospital, Kiinamyllynkatu 4–6, P.O. Box 52, 20521 Turku, Finland; 2 Turku Bioscience Centre, University of Turku and Åbo Akademi University, Tykistökatu 6, 20520 Turku, Finland; 3 Department of Medical Physics, Turku University Hospital, Turku, Finland; 4 Institute of Biomedicine, University of Turku, Turku, Finland; 5 InFLAMES Research Flagship Center, University of Turku, Turku, Finland

**Keywords:** Rheumatic diseases, Risk factors, Computational models

## Abstract

Frequent laboratory monitoring is recommended for early identification of toxicity when initiating conventional synthetic disease-modifying antirheumatic drugs (csDMARDs). We aimed at developing a risk prediction model to individualize laboratory testing at csDMARD initiation. We identified inflammatory joint disease patients (N = 1196) initiating a csDMARD in Turku University Hospital 2013–2019. Baseline and follow-up safety monitoring results were drawn from electronic health records. For rheumatoid arthritis patients, diagnoses and csDMARD initiation/cessation dates were manually confirmed. Primary endpoint was alanine transaminase (ALT) elevation of more than twice the upper limit of normal (ULN) within 6 months after treatment initiation. Computational models for predicting incident ALT elevations were developed using Lasso Cox proportional hazards regression with stable iterative variable selection (SIVS) and were internally validated against a randomly selected test cohort (1/3 of the data) that was not used for training the models. Primary endpoint was reached in 82 patients (6.9%). Among baseline variables, Lasso model with SIVS predicted subsequent ALT elevations of > 2 × ULN using higher ALT, csDMARD other than methotrexate or sulfasalazine and psoriatic arthritis diagnosis as important predictors, with a concordance index of 0.71 in the test cohort. Respectively, at first follow-up, in addition to baseline ALT and psoriatic arthritis diagnosis, also ALT change from baseline was identified as an important predictor resulting in a test concordance index of 0.72. Our computational model predicts ALT elevations after the first follow-up test with good accuracy and can help in optimizing individual testing frequency.

## Introduction

Routine laboratory monitoring is recommended for early identification of toxicity during treatment with conventional synthetic disease modifying antirheumatic drugs (csDMARDs). Currently, multiple national laboratory monitoring recommendations exist^1–5^. Most of these recommendations, which are based on literature reviews and expert consensus, suggest monitoring for toxicity every 2–4 weeks for the first 3 months and 1–3 monthly thereafter. Overly frequent monitoring should be avoided as it causes burden for patients and healthcare systems. However, data on optimal timing of monitoring remains elusive.

The 2017 British Society for Rheumatology guideline for monitoring non-biologic DMARD therapy recommendations advise that laboratory tests should be taken every 2 weeks when initiating a new csDMARD until on a stable dose for 6 weeks, and quarterly thereafter^5^. In addition, more frequent monitoring should be considered for patients at high risk for toxicity. In general, most recommendations advocate measuring a minimum of complete blood count (CBC), creatinine or calculated glomerular filtration rate, and alanine amino transferase (ALT) at baseline and at follow-up^1–3,5^.

Previous studies addressing safety monitoring have focused on methotrexate. Busger et al. compared the influence of monitoring on methotrexate survival in psoriasis and psoriatic arthritis patients^6^. They found that more frequent monitoring, recommended for psoriasis by dermatologists, led to reduced drug survival without differences in severe adverse events compared to a less stringent monitoring strategy. Malley et al. studied inflammatory arthritis patients on stable dose of methotrexate alone or in combination with other DMARDs, and Karlsson Sundbaum et al. and Schmajuk et al. focused on risk factors for ALT elevation in users of methotrexate^7–9^. Two latter studies recognized pre-treatment ALT elevation as a risk factor for later elevations. Other previously identified risk factors for ALT elevation in methotrexate users include obesity^7,10,11^, high cholesterol, and lack of folic acid supplementation^7,10^. Findings on rheumatoid arthritis (RA) seropositivity, biologic DMARDs, statins, and female sex have been inconsistent^7,9,12^. However, defining which patients could be monitored less often is difficult based on previous literature.

In the current study, we aimed to develop a computational model using electronic health record (EHR) data for prediction of ALT elevations at csDMARD initiation. Our second aim was to evaluate the incidence of laboratory abnormalities, with special interest in ALT elevations, in patients who initiated 1–2 csDMARDs in routine rheumatology clinical practice.

## Materials and methods

### Study sample

We identified patients aged at least 18 years with inflammatory joint disease who visited the rheumatology clinic of Turku University Hospital in 2013–2019 and were prescribed a new csDMARD course requiring laboratory monitoring (methotrexate with folic acid supplementation, sulfasalazine, leflunomide or azathioprine) using EHR data. Dates of birth and death were drawn from the EHR data together with baseline and follow-up safety monitoring results. After csDMARD initiation, data on a minimum of three laboratory tests (baseline and two follow-up tests including CBC values and ALT) during the first 6 months were required for confirming an initiated csDMARD course and cohort inclusion. For rheumatoid arthritis patients diagnoses (clinical diagnosis by treating rheumatologist) and csDMARD initiation and cessation dates were also manually confirmed from the EHR. Finally, all patients were randomly divided in a 2:1 ratio into two cohorts: training cohort for model development and test cohort for internal validation. This retrospective register-based study was approved by the institutional review board of Turku University Hospital (T86/2019) and conducted in accordance with the Declaration of Helsinki. Due to the retrospective study design, informed patient consent was waived by the institutional review board of Turku University Hospital.

### Reference ranges for laboratory monitoring and study endpoints

Toxicity events, defined as laboratory values beyond nationally determined decision/action limits^4^, i.e. values requiring rheumatology team consultation or interruption in treatment, at any time point during the first 6 months after treatment initiation, were considered as primary outcomes of interest. The following limits for toxicity events were applied: white blood cell count (WBC) < 3.0 × 10^9^/l, absolute neutrophil count (ANC) < 1.0 × 10^9^/l, platelet count < 100 × 10^9^/l, and ALT > 2 × upper limit of normal (ULN; i.e. > 2 × 50 U/l = 100 U/l for men and > 2 × 35 U/l = 70 U/l for women). Creatinine values were measured, as recommended by national guidelines^4^, at baseline and at 6 months in most patients. Therefore, data on creatinine values during the follow-up were scarce and not included in the analyses.

### Statistical analyses and development of prediction models for toxicity events

For the prediction of each toxicity event, we considered age, sex, diagnosis, used csDMARDs as well as baseline laboratory values used for defining the event as candidate predictors. To explore the initial regression coefficients before penalization with Lasso regression and to provide easily interpretable estimates of relative risk as hazard ratios (HRs), we performed multivariable Cox proportional hazards regression analysis with all candidate predictors included in the model. Additionally, we performed unadjusted analyses to demonstrate the relative risks in the case when only a single variable would have been used for risk prediction. In the comparisons, female sex, a diagnosis of seropositive RA, or the use of methotrexate as the primary treatment regimen were used as reference groups due to being the largest groups in our study population. Patients with a toxicity event at baseline were excluded from the analyses. Additionally, time from beginning of the treatment to first toxicity event was visualized using the Kaplan–Meier estimator for selected variables of interest and compared between different patient groups using the log-rank test. In survival analyses, patients who did not reach 6-month follow-up time point were censored at potential date of death, csDMARD cessation, or date of data extraction [April 30th 2019 for RA patients and December 31st 2019 for patients with other inflammatory joint disease (IJD)], whichever occurred first.

Besides the baseline laboratory values, we also used the result of the first follow-up test as an explanatory variable for later toxicity events. We considered the rate of change, defined as change in test result with respect to baseline, divided by time elapsed, as an additional explanatory variable and repeated the search for potential risk factors. In these analyses, only patients without a toxicity event at baseline or first follow-up point were included.

To develop computational models for predicting the probability of surviving 180 days of csDMARD therapy without laboratory abnormalities, we applied Lasso penalized Cox proportional hazards regression together with stable iterative variable selection (SIVS) to the data in the training cohort to identify the most influential variables on the outcome and to develop a simple-to-use computational model for clinical use^13^. Previously, penalized regression with SIVS has been reported to be an effective method for developing accurate, well-generalizable models with minimum number of variables^14,15^. In SIVS, model training with fivefold cross-validation was repeated 100 times. Discrimination performance of the models was reported for the internal validation cohort in terms of Harrell's concordance index using the final model coefficients derived from training data.

All statistical analyses and modelling were carried out using the R statistical computing environment version 4.0.3 (R Core Team, 2016. R: A language and environment for statistical computing. R Foundation for Statistical Computing, Vienna, Austria. URL https://www.R-project.org/). R packages survival (version 3.2-10)^16^, ggplot2 (version 3.3.3)^17^, sivs (version 0.2.5) and glmnet (version 4.1-1)^18^ were used for time-to-event analysis, visualization of results, variable selection and penalized regression, respectively. The level of significance was set at *P* < 0.05.

## Results

**Figure 1:**
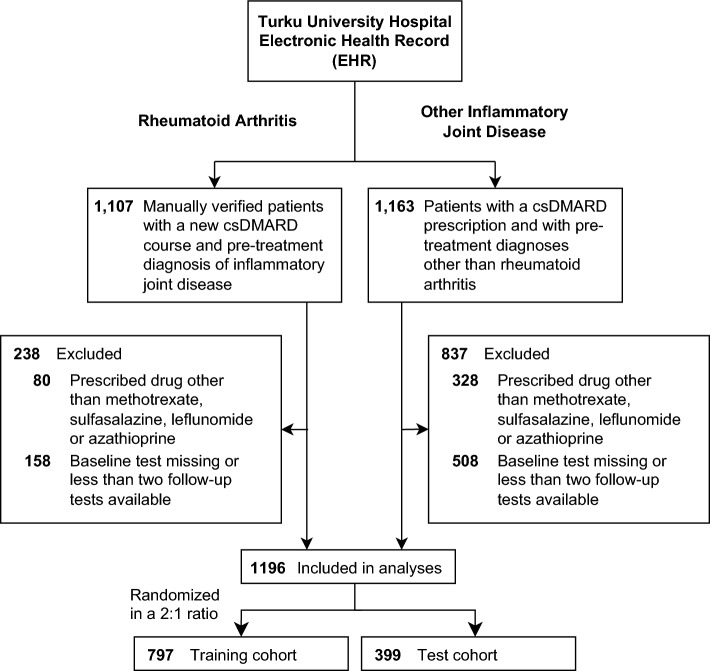
Selection of patients.

We identified 1541 patients with a diagnosis of RA and a new csDMARD course from the Turku University Hospital EHR. Diagnosis and initiation of a csDMARD was manually confirmed in 1107 RA patients. Of these, 869 who initiated a pre-specified csDMARD and had a minimum of three safety monitoring tests available (Fig. 1). Additionally, using the same criteria, we added 327 of the 1163 patients extracted directly from the EHR, with inflammatory joint disease other than RA, to the overall patient cohort (Fig. 1). Finally, all included patients were randomly divided into separate training (797 patients) and test (399 patients) cohorts for model development and internal validation, respectively. Baseline characteristics of the cohorts are presented in Table 1.

**Table 1:** Baseline characteristics of the study population according to cohort type.

Characteristic	Training cohort (N = 797)	Test cohort (N = 399)	*P* value
Age, median (range)	57 (18–93)	58 (18–91)	0.79
Sex, N (%)			0.80
Men	291 (36.5)	142 (35.6)	
Women	506 (63.5)	257 (64.4)	
Diagnosis, N (%)			0.47
Seropositive RA	388 (48.7)	179 (44.9)	
Seronegative RA	192 (24.1)	110 (27.6)	
Psoriatic arthritis	65 (8.2)	39 (9.8)	
Axial spondyloarthritis	62 (7.8)	26 (6.5)	
Unspecified arthritis	55 (6.9)	25 (6.3)	
Reactive arthritis	24 (3.0)	15 (3.8)	
Juvenile idiopathic arthritis	8 (1.0)	4 (1.0)	
Inflammatory bowel disease associated arthritis	3 (0.4)	1 (0.3)	
Laboratory test result, median (IQR)			
White blood cell count [× 10^9^/l]	7.6 (6.2–9.4)	7.3 (6.2–9.1)	0.24
Platelet count [× 10^9^/l]	288 (245–346)	289 (242–343)	0.82
Absolute neutrophil count [× 10^9^/l]	4.7 (3.3–6.2)	4.4 (3.3–5.8)	0.09
Alanine amino transferase [U/l]	20.0 (15.0–29.0)	21.0 (15.0–29.0)	0.60
csDMARDs, N (%)			0.57
Methotrexate (MTX)	357 (44.8)	183 (45.9)	
Sulfasalazine (SSZ)	207 (26.0)	113 (28.3)	
Azathioprine (AZA)	21 (2.6)	14 (3.5)	
Leflunomide (LEF)	36 (4.5)	20 (5.0)	
Combination therapy (MTX + SSZ)	172 (21.6)	68 (17.0)	
Combination therapy (AZA/LEF + SSZ)	4 (0.5)	1 (0.3)	

Comparisons between the cohorts were tested using the Mann–Whitney test for continuous variables and the chi-squared test or Fisher’s exact test (N < 5) for categorical variables.

*RA* rheumatoid arthritis, *IQR* interquartile range, *csDMARD* conventional synthetic disease-modifying antirheumatic drug.

Although the majority of the patients’ baseline laboratory values were within the reference range, we identified several patients with abnormal values at baseline. In the overall patient cohort, 4 patients had low WBC, 3 patients low ANC, 4 patient low platelets, and 19 patients elevated ALT at baseline.

The most common laboratory abnormality during the follow-up was elevated ALT (Table 2). In the training cohort, 56 (7.0%) patients, and respectively in the test cohort 26 (6.5%) patients, experienced an ALT elevation at follow-up. Furthermore, in 31 of 54 (57%) ALT elevations among RA patients, including four patients initiating two csDMARDs, elevated ALT led to csDMARD cessation during the first 6 months. For the patients with IJD other than RA, we present no data on medication cessations as they were not manually confirmed. Abnormalities in other laboratory tests were rare (occurrence < 1.0%). Therefore, we focused only on evaluating risk factors and developing prediction models for ALT elevations in the subsequent analyses. Per patient a median of four follow-up tests were available (Table 2).

**Table 2:** Number of follow-up laboratory monitoring tests performed during the first 6 months after treatment initiation and number of detected alanine amino transferase elevations of more than twice the upper limit of normal (toxicity events).

Laboratory test	Training cohort (N = 797)					Test cohort (N = 399)				
	Tests total	Tests per patient		Patients with toxicity event*		Tests total	Tests per patient		Patients with toxicity event*	
		Median	(range)	N	(%)		Median	(range)	N	(%)
White blood cell count (WBC)	3429	4	(2–25)	4	(0.5)	1629	4	(2–22)	1	(0.3)
Platelet count	3428	4	(2–25)	2	(0.3)	1626	4	(2–22)	1	(0.3)
Absolute neutrophil count (ANC)	3283	4	(2–14)	1	(0.1)	1573	4	(2–12)	3	(0.8)
Alanine amino transferase (ALT)	3371	4	(2–26)	56	(7.0)	1586	4	(3–26)	26	(6.5)

*Toxicity events were defined using the following reference ranges: WBC < 3.0 × 10^9^/l, ANC < 1.0 × 10^9^/l, platelet count < 100 × 10^9^/l, and ALT > 2 × reference range (i.e. > 2 × 50 U/l = 100 U/l for men and > 2 × 35 U/l = 70 U/l for women). Patients with toxicity detected at baseline were excluded from the analysis.

In the initial Cox proportional hazards regression analysis, of the baseline variables, ALT [unadjusted hazard ratio (HR) 7.60 per increase by sex-specific reference range, 95% confidence interval (CI) 4.26–13.6, *P* < 0.001; adjusted HR 7.01 per increase by sex-specific reference range, 95% CI 3.81–12.9, *P* < 0.001], a diagnosis of psoriatic arthritis [unadjusted HR 3.73, 95% CI 1.82–7.62, *P* < 0.001; adjusted HR 2.70, 95% CI 1.20–6.07, *P* = 0.02] and initiating a csDMARD course other than methotrexate, sulfasalazine, or their combination [adjusted HR 2.81, 95% CI 1.24–6.37, *P* = 0.01], were associated with incident ALT-elevations (Table 3). In addition to these, greater rate of ALT change from baseline to first follow-up test was associated with subsequent ALT elevations in both univariable [HR 2.12 per change in normalized ALT (with respect to sex-specific reference range) per 30 days, 95% CI 1.34–3.35, *P* = 0.001] and multivariable analyses (HR 2.83 per change in normalized ALT per 30 days, 95% CI 1.80–4.42, *P* < 0.001) for ALT elevations occurring after the first follow-up test (Table 3).

**Table 3:** Associations of potential explanatory baseline and first follow-up variables and subsequent alanine amino transferase (ALT) elevations of more than twice the upper limit of normal (ULN) in the training cohort consisting of rheumatoid arthritis patients using univariable and multivariable Cox proportional hazards regression.

Explanatory variables	Unadjusted			Adjusted		
	HR	(95% CI)	*P* value	HR	(95% CI)	*P* value
At baseline (N = 860)						
Age (per year)	1.00	(0.98–1.01)	0.70	1.01	(0.99–1.03)	0.59
Sex (male)	0.71	(0.39–1.29)	0.26	0.75	(0.41–1.40)	0.37
Baseline ALT*	7.60	(4.26–13.6)	< 0.001	7.01	(3.81–12.9)	< 0.001
Diagnosis						
Seropositive RA	Ref	–	–	–	–	–
Seronegative RA	1.02	(0.48–2.19)	0.95	1.01	(0.47–2.20)	0.97
Psoriatic arthritis	3.73	(1.82–7.62)	< 0.001	2.70	(1.20–6.07)	0.02
Other inflammatory joint disease or unspecified arthritis	1.22	(0.57–2.61)	0.60	1.27	(0.55–2.95)	0.58
csDMARDs						
Methotrexate (MTX)	Ref	–	–	Ref	–	–
Sulfasalazine (SSZ)	0.83	(0.42–1.65)	0.59	1.11	(0.53–2.34)	0.78
MTX and SSZ	0.58	(0.25–1.34)	0.20	0.78	(0.32–1.88)	0.57
Other^§^	2.08	(0.94–4.62)	0.07	2.81	(1.24–6.37)	0.01
After 2nd test (N = 831)						
Age (per year)	1.00	(0.98–1.01)	0.65	1.00	(0.98–1.02)	0.80
Sex (male)	0.48	(0.22–1.06)	0.07	0.42	(0.19–0.97)	0.04
Baseline ALT*	4.56	(2.09–9.96)	< 0.001	6.42	(2.64–15.63)	< 0.001
ALT rate of change**	2.12	(1.34–3.35)	0.001	2.83	(1.80–4.42)	< 0.001
Diagnosis						
Seropositive RA	Ref	–	–	Ref	–	–
Seronegative RA	0.77	(0.30–1.96)	0.58	0.69	(0.26–1.85)	0.47
Psoriatic arthritis	3.20	(1.37–7.48)	0.007	2.86	(1.12–7.34)	0.03
Other inflammatory joint disease or unspecified arthritis	1.09	(0.45–2.64)	0.86	1.15	(0.42–3.12)	0.78
csDMARDs						
Methotrexate (MTX)	Ref	–	–	Ref	–	–
Sulfasalazine (SSZ)	0.77	(0.34–1.77)	0.54	1.18	(0.47–2.93)	0.73
MTX and SSZ	037	(0.21–1.54)	0.27	0.80	(0.28–2.26)	0.67
Other^§^	2.32	(0.92–5.85)	0.07	3.81	(1.45–10.00)	0.007

*Normalized according to sex-specific reference range (35 U/l for women, 50 U/l for men).

**Change in normalized ALT values between the baseline and the first follow-up measurement scaled by time elapsed (in months). Patients with ALT > 2 × ULN at baseline were excluded from the baseline analysis (9 patients) and respectively, patients with ALT > 2 × ULN at baseline or at first follow-up were excluded from the analysis using baseline and first follow-up data (38 patients). Overall, 50 toxicity events were detected at follow-up. At first follow-up, a total of 34 toxicity events remained to be detected at later follow-up measurements.

^§^Other csDMARDs; azathioprine or leflunomide alone or in combination with sulfasalazine.

**Figure 2:**
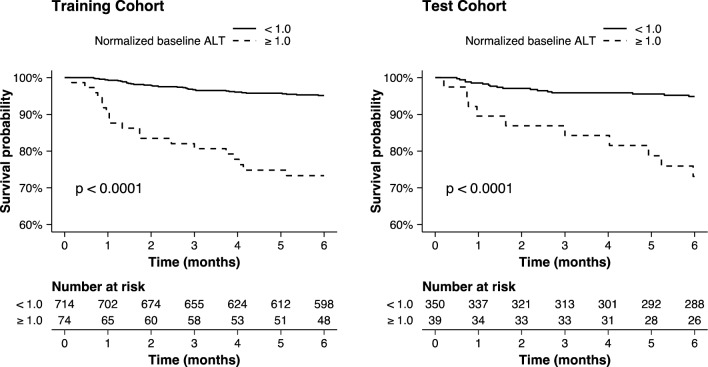
Kaplan–Meier survival curves for alanine amino transferase (ALT) elevations of more than twice the upper limit of normal grouped according to normalized baseline ALT. The normalization was done with respect to sex-specific reference range (35 U/l for women, 50 U/l for men). Patients with elevated ALT at baseline were excluded from the analysis.

By applying Lasso penalized Cox proportional hazards regression to the baseline data, ALT, psoriatic arthritis diagnosis, and the use of csDMARD other than methotrexate or sulfasalazine were identified as the strongest predictors of subsequent ALT elevations reaching together a concordance index of 0.71 (95% CI 0.60–0.82) in the test cohort. From the initial regression coefficients, the corresponding risk equation was reduced to

$$Survival \,\left( \% \right) = 0.954^{{\exp \left( {1.900 \times ALT_{0} + 0.824 \times Diag_{PsA} + 0.985 \times csDMARD_{other} - 1.110} \right)}} \times 100\%$$

where *ALT*_0_ indicates normalized baseline ALT, ${Diag}_{PsA}$ is a binary indicator for psoriatic arthritis diagnosis (0 if no, 1 if yes), and $csDMAR{D}_{other}$ is a binary indicator for csDMARD group (0 if methotrexate, sulfasalazine or their combination, 1 otherwise). Among all selected predictors, ALT level was the most influential one and we observed that patients with moderately elevated baseline ALT (1–2 × ULN) had significantly higher incidence of significant ALT elevations (> 2 × ULN) during follow-up compared to patients with normal baseline ALT (Fig. 2).

**Figure 3:**
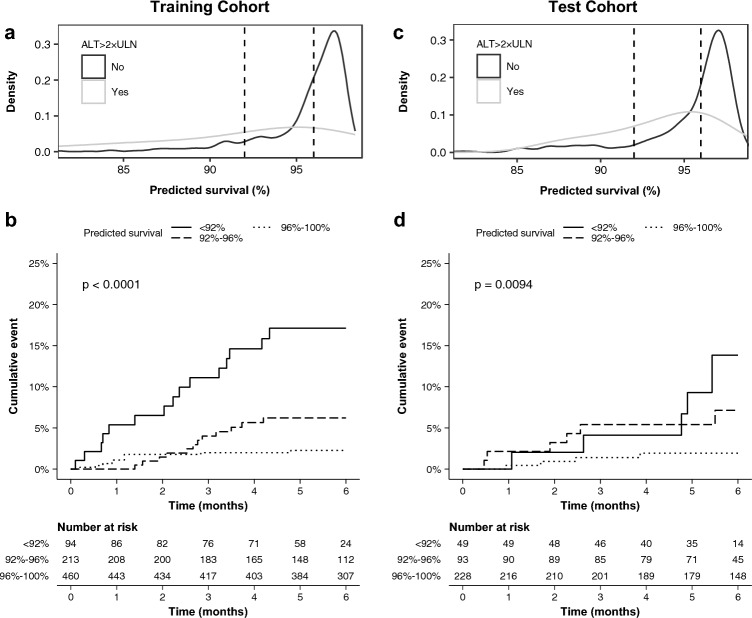
Evaluation of survival estimates for treatment without elevated alanine amino transferase (ALT) obtained using the Lasso penalized Cox proportional hazards regression model trained using data from baseline and the first follow-up measurement. Using the survival estimates (**a**,**c**), we stratified patients into groups (**b**,**d**) based on predicted probability (< 92%, 92–96%, > 96%) to observe the cumulative incidence of ALT elevations during the following 180 days of conventional synthetic disease-modifying anti-rheumatic drug treatment.

For events occurring after the first follow-up test, we repeated the model development process with additional input data and Lasso regression identified also change in ALT as an important predictor instead of csDMARDs, reaching slightly higher internal validation AUROC of 0.72 (95% CI 0.59–0.84) in the test cohort. The obtained risk equation for later ALT elevations was

$$Survival \,\left( \% \right) = 0.962^{{\exp \left( {1.709 \times ALT_{0} + 0.834 \times \frac{{ALT_{1} - ALT_{0} }}{t} + 0.971 \times Diag_{PsA} - 1.029} \right)}} \times 100\%$$

where *ALT*_0_ indicates normalized baseline ALT, *ALT*_1_ is normalized follow-up ALT, *t* time elapsed between baseline and follow-up tests (in months), and $Diag_{PsA}$ is a binary indicator for psoriatic arthritis diagnosis (0 if no, 1 if yes). The predicted survival estimates obtained in the training cohort using this equation ranged between 80 and 98% with median value of 96% (Fig. 3). After stratification, if the survival estimate was over 96%, the observed cumulative incidence of ALT elevations was only 2.3% in the training cohort and 1.8% in the test cohort. In contrast, if the survival estimate was lower than 96%, likelihood of ALT elevations was substantially higher and increased towards lower survival estimates.

## Discussion

In the current study, patients with low normal ALT at baseline and no rising trend at first follow-up were unlikely to develop significant liver toxicity during the first 6 months of csDMARD treatment. Compared to other csDMARDs, the risk for incident ALT elevations was lower among users of methotrexate and sulfasalazine. Increased risk was observed in patients with psoriatic arthritis. Other laboratory abnormalities were rare. We created a simple computational model for predicting ALT elevations, which can be used for individual optimization of laboratory testing during csDMARD initiation.

In our analyses, predictions effectively reflected the occurrence of ALT elevations suggesting that individualization of monitoring is possible. This challenges most guidelines, which suggest safety monitoring of csDMARD treatment every 2–4 weeks for the first 3 months and every 4–12 weeks thereafter^1,2,5^. Based on our results, more intensive monitoring could be targeted at patients with elevated ALT (or other laboratory abnormalities) at baseline or a rising trend at first follow-up after reaching the desired csDMARD dose. Based on our own experience less frequent monitoring is likely sufficient for most patients. Since reaching a national consensus in 2015, monitoring intervals at csDMARD initiation were prolonged from biweekly to 3, 6 and 12 weeks in Finland^4^. In these national guidelines, ALT-elevations above 2–2.5 times the sex-specific ULN were set as the decision limit for temporary DMARD pause or cessation for health care professionals. Some clinicians may, however, use lower, individualized ALT-thresholds. So far, no signals suggesting a significant increase in severe adverse events have emerged. Admittedly, less frequent testing likely leads to lower number of detected mild, intermittent ALT elevations. Most of these normalize without change in csDMARD treatment and are unlikely to cause harm to patients. In contrast, constant ALT elevations are usually seen before development of significant liver toxicity^19^. These will be detected despite less frequent monitoring.

We found that elevated baseline ALT and rising trend at first follow-up were associated with increased risk of ALT elevations. This is in line with previous studies, which have found elevated baseline ALT to be the strongest predictor of future ALT elevations during methotrexate treatment^7,9,12^. Further, we found an association between a diagnosis of psoriatic arthritis and ALT-elevations. Obesity and non-alcoholic fatty liver disease (NAFLD) are common comorbidities in patients with plaque psoriasis and psoriatic arthritis and may potentially explain the increased risk of ALT-elevations^20–22^. Obesity has also been recognized as a risk factor for incident psoriasis^21^. Recent research has suggested that psoriasis and NAFDL may even share common disease pathways^23^. Unfortunately, we were not able to adjust the analyses for weight due to lack of reliable EHR data to study these associations further. We did not find associations between ALT elevation and anti-citrullinated antibody positivity^12^ or female sex^7,9^, and had no reliable EHR data on other possible risk factors like high cholesterol^7^, and statin treatment^7,9^. We also found that patients initiating other csDMARDs than methotrexate, sulfasalazine or their combination were at higher risk for ALT elevation. Although methotrexate and sulfasalazine are generally well tolerated, the observed risk reduction may be explained by a selection bias and should be confirmed in other cohorts. Methotrexate alone (or in combination with sulfasalazine) is usually the first-line treatment of RA and respectively, sulfasalazine is used commonly in axial spondyloarthritis. In contrast, leflunomide and azathioprine are often prescribed as second- or third-line options for patients who have contraindications or intolerance to first line treatments and may thus be already more prone to toxicity.

Prevalence of significant ALT elevations (> 2 × ULN) during the 6-month follow-up was 6.9% in the overall patient cohort, which is in accordance with previous studies. A 2015 meta-analysis of 32 clinical trials found mild liver enzyme elevations (< 3 × ULN) in 7.9% and respectively, significant elevations (> 3 × ULN) in 3.3% of patients^24^. In the largest study of low-dose methotrexate associated adverse events, ALT elevations (> 2 × ULN) were detected in 10.7% of 2391 patients with increased cardiovascular risk and no IJD over 3 years^25^. Previous studies suggest that extending monitoring intervals in long-term treatment is a presumably safe alternative for select patients noting that persistent liver enzyme elevations are most likely detected despite less frequent monitoring^7–9^. In addition, we suggest more individual monitoring already at treatment initiation based on predicted risk of ALT elevation. Our simple computational model is well suited for identifying patients at high risk for liver toxicity.

Our study has limitations, like the retrospective single-center design. However, our cohort of inflammatory joint disease patients from seven consecutive years represents a typical rheumatology outpatient clinic patient population, making our results generalizable to everyday clinical practice. It should be noted that, for patients with IJD other than RA, we used data drawn directly from EHR without ascertainment of diagnoses and medication initiation/cessation dates. Further, reliable data on patients’ complete medication, weight and previous medical history were not available and therefore not included in the analyses, which is a possible cause of bias. However, we chose to use only a limited number of variables and aimed at creating a simple, EHR data based prediction model that can be easily instituted to everyday clinical practice. Despite above mentioned factors, predictions in the internal validation cohort had good accuracy, suggesting sufficient data quality. Taking these limitations into account, we suggest that the reliability of our prediction model should be confirmed in a larger cohort.

In summary, we found that most ALT elevations during the first 6 months of csDMARD treatment can be predicted based on two first safety monitoring tests. In patients initiating a csDMARD course, other laboratory abnormalities were rare. We suggest that clinicians may consider individualized testing strategies after the first follow-up test based on predicted risk of ALT elevations.

## Data Availability

The Finnish law does not allow open sharing of the data used in this study, but data access is possible via formal material transfer agreements (MTA). Investigators that wish to access the data are encouraged to contact the corresponding author.
